# Role of the infectious disease consultant in improving antimicrobial therapy prescription in neurosurgery

**DOI:** 10.1186/1753-6561-5-S6-P154

**Published:** 2011-06-29

**Authors:** L Pagani, R Aschbacher, PC Cecchi, S Vidoni, G Spoladore, P Mian, A Schwarz

**Affiliations:** 1 Bolzano Central Hospital, Unit for Hospital Antimicrobial Chemotherapy, Bolzano, Italy; 2 Bolzano Central Hospital, Neurosurgery Unit, Bolzano, Italy

## Introduction / objectives

To assess whether a systematic infectious disease consultation programme (IDCP) contributes to improve the diagnostic and therapeutic approach to infected patients in a 21-bed neurosurgery (NS) unit of an Italian 850-bed tertiary care centre.

## Methods

The IDCP was based on regular consultations by the ID specialist for practice recommendations of prophylaxis or targeted therapy prescriptions, driven by local antimicrobial resistance surveillance and clinical pharmacology parameters, together with antibiotic formulary restrictions; the activity also included weekly reassessment and feedback meetings with NS staff, and subsidiary telephone calls; all the activities were recorded in electronic databases. The ICPC started in 2008; data related to the ID consultations, microbiological sampling and antibiotic prescriptions including costs were analyzed for one year before (2007) and two years after the ICPC implementation (2008 and 2009).

## Results

ID consultations were 134, 125 and 154 in 2007, 2008, 2009 respectively. In particular, consultations to initiate empiric treatment and to either adjust or streamline ongoing therapy increased significantly from 2007 to 2009. Annual expenditures for antimicrobials decreased from € 88,800 in 2007, to € 60,500 in 2008, and to € 49,000 in 2009. From 2007 to 2009, the annual number of collected microbiological specimens significantly decreased (1829, 1031, 970, respectively).

## Conclusion

The introduction of an IDCP in the NS unit improved the appropriateness of microbiological diagnostic sampling and antimicrobial therapy prescription and led to significant reduction of costs attributable to antimicrobials.

## Disclosure of interest

None declared.

